# Experimental Evaluation of Advertisement-Based Bluetooth Low Energy Communication

**DOI:** 10.3390/s20010107

**Published:** 2019-12-23

**Authors:** Maciej Nikodem, Marek Bawiec

**Affiliations:** Department of Computer Engineering, Faculty of Electronics, Wrocław University of Science and Technology, Wybrzeże Wyspiańskiego 27, 50-370 Wrocław, Poland; marek.bawiec@pwr.edu.pl

**Keywords:** Bluetooth Low Energy, advertising mode, Internet of Things, large-scale system, system validation, experimental evaluation

## Abstract

This paper addresses the efficiency of Bluetooth Low Energy (BLE) communication in a network composed of a large number of tags that transmit information to a single hub using advertisement mode. Theoretical results show that the use of advertisements enables hundreds and thousands of BLE devices to coexist in the same area and at the same time effectively transmit messages. Together with other properties (low power consumption, medium communication range, capability to detect a signal’s angle-of-arrival, etc.), this makes BLE a competing technology for the Internet of Things (IoT) applications. However, as the number of communicating devices increases, the advertisement collision intensifies and the communication performance of BLE drops. This phenomena was so far analyzed theoretically, in simulations and in small-scale experiments, but large-scale experiments are not presented in the literature. This paper complements previous results and presents an experimental evaluation of a real IoT-use case, which is the deployment of over 200 tags communicating using advertisements. We evaluate the impact of the number of advertisements on the effective data reception rate and throughput. Despite the advertisement collision rate in our experiment varying between 0.22 and 0.33, we show that BLE, thanks to the multiple transmission of advertisements, can still ensure acceptable data reception rates and fulfill the requirements of a wide range of IoT applications.

## 1. Introduction

**Figure 1:**
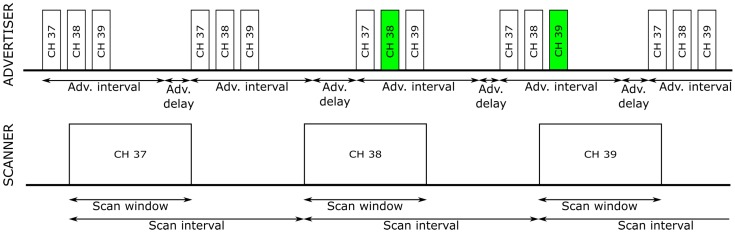
Bluetooth Low Energy (BLE) advertisement and scanner operation in connectionless mode. The green color denotes advertisements that are coincidentally received, i.e., when the hub happens to scan the same channel as the one the tag uses for transmission.

Bluetooth Low Energy (BLE) is a widespread communication protocol that is getting more and more interest as a technology for Internet of Things (IoT) solutions. BLE has been developed for several years now, with version 5.1 being published in early 2019. BLE has two modes of operation: connectionless (also called advertisement) and connected (also called paired). Connectionless operation uses advertisement messages and optional scan request–response message pairs to transmit data from end-devices (also called advertisers or broadcasters) to a central device (also called a scanner or a hub). This is a one-way broadcast communication without acknowledgments (Figure 1), which means that data is only transferred from end-devices to scanners.

Advertisers broadcast messages repeatedly (every *advertisement interval* plus random *advertisement delay*) using three (out of a total of 40) shared radio channels (numbered 37, 38, and 39). The advertisement interval is between 20 ms and 10.24 s, and the advertisement delay is a random value in the range of 0 to 10 ms. Scanners continuously scan these channels in order to receive transmissions coincidentally (scanners switch channels every *scan interval* and listen for the advertisements for *the scan window* on every channel, see Figure 1). Upon reception of the advertisement, the scanner may send a scan request message that is answered with a scan response, which in turn contains additional information.

When the scanner receives the advertisement from the end-device it can initiate a connection (pairing) and establish a link that enables bidirectional data transmission. While the connection is active, a dedicated communication channel between both devices is established and used for data exchange. In the connected mode, BLE devices use dedicated radio channels (out of a total of 37 data channels available), avoiding channels that are used by neighboring devices, and switching channels to ensure coexistence with other 2.4 GHz radios in the vicinity.

BLE connection-based communication is typically used in small, embedded devices in a wide range of different applications such as wearables, smart sensors (e.g., [1]), healthcare monitoring systems (e.g., [2]), home automation and smart homes, and indoor positioning (e.g., [3]). In these applications, sensor data is exchanged after a connection is established, and advertisements are only used by the central device (e.g., a mobile phone or hub) to detect sensors. As the connected mode has been widely used and demonstrated in various applications, it has been thoroughly evaluated and tested, among others, for the following features: throughput, latency, energy consumption [4,5,6], coexistence with other BLE devices, as well as other radio technologies operating in the same radio band: WiFi, ZigBee, and IEEE 802.15.4 [7,8,9]. From the vast number of network deployments and various published research results, BLE can be considered as a well designed communication technology that is robust and reliable, even in the highly occupied 2.4 GHz spectral band. However, the number of simultaneous connections is limited by the number of available radio channels and available resources in the device [5] (e.g., 8 for STMicroelectronics tags and 20 for Nordic Semiconductor tags). Consequently, the connected mode does not scale well and its application to large scale IoT systems is limited.

With the introduction of the IoT concept, more focus has been put on radio technologies that enable the communication of thousands of end-devices to the network. This led to the development of new communication technologies (e.g., sub-GHz) and introduced new network communication concepts, where high capacity, the ability to serve thousands of end-devices, energy efficiency, and autonomous operation are more important than communication speed, channel throughput, or complex radio topologies. Using advertisements for data communication in connectionless BLE is similar to these approaches, making connectionless operation attractive for IoT applications.

Compared to connection-based communication, the connectionless mode has several limitations, which primarily come from using advertisement and optional scan response messages for data transmission. These limitations include a small payload size, relatively long transmission intervals (affecting effective data throughput and transmission delay), a small number of radio channels used (thus increased probability of collisions), and a lack of acknowledgments to confirm that the transmission was correctly received [10,11,12]. Although these form a limitation, it is worth noticing that several communication technologies and protocols used in IoT networks (e.g., LoRaWAN, SigFox) do not provide the same properties and can still be effectively used in a vast number of applications. Furthermore, there are some aspects that suggest that connectionless BLE, when compared to WiFi, ZigBee/IEEE 802.15.4, and even sub-GHz solutions, can be better suited for small-to-medium network size IoT applications. Firstly, it does not have the scaling issue, which means that theoretically it can effectively communicate data between thousands of devices, and is often proposed as a method for opportunistic data transmission [13,14], monitoring applications [15], and localization systems based on radio signal strength [3,16]. BLE outperforms other 2.4 GHz technologies in terms of energy costs, throughput, and latency [7,17]. Compared to sub-GHz technologies, BLE has no duty-cycling restrictions, which limit the amount of time a radio can use for transmission. Moreover, the energy efficiency [18] and expected lifetime [19] of BLE outperforms unlicensed sub-GHz technologies. BLE is also much more widespread, and the newest version of the standard (BLE 5.1) enables the receiver to estimate an angle-of-arrival, which can be used for accurate localization of communicating devices and further extend the range of possible applications. Additionally, in the connectionless mode, scan request and scan response messages are optional and do not need to be used. This means that the tag may not receive any radio packets and may turn off the radio after transmission, as a consequence, reducing energy consumption. Moreover, the connectionless mode does not incur any additional energy cost required to establish and maintain the connection. This provides further energy savings. Consequently, connectionless BLE is an attractive technology for IoT applications, where a large number of devices coexist in a small or medium size area.

Taking the above into account, real-life evaluation of connectionless BLE communication becomes important, especially since the results presented in the literature are mainly based on simulations and theoretical analyses addressing device discovery latency (e.g., [11,12]), the coexistence of a small number of devices [20], and energy efficiency [5,10,13,21,22]. The contribution of this paper includes the following:

- Experimental evaluation of a large number of BLE devices communicating simultaneously using advertisement messages in the presence of mutual and external interference;
- Demonstration of the applicability of the connectionless BLE mode to large-scale and medium-area IoT applications.

The paper is organized as follows. Section 2 presents related work and previous results of BLE advertisement mode evaluation. Section 3 describes our evaluation setup, scenarios, and metrics used in the experiments. Section 4 presents the results of our experiments and discusses the performance of BLE connectionless communication for different settings of the parameters. The paper is summarized in Section 5, which draws conclusions and discusses the applicability of the BLE advertisement mode to large-scale IoT applications.

## 2. Related Work

Aguilar et al. [13] analyzed the performance of both connection-based and connectionless BLE communication for opportunistic data collection from sensors. For connected mode, they assumed that there are only two BLE devices in the network so there was no need for the central device to switch between the end-devices. As a result, consequences of switching (e.g., on throughput) were not taken into account. Theoretical analyses focused on the evaluation of the end-device’s current consumption, energy cost per bit, lifetime, and maximum amount of data transmitted. The experimental evaluation was only conducted for connected communication of two BLE devices. The presented results show that connectionless communication has a significantly lower current consumption and is less affected by transmission errors, but at the same time allows less data to be transmitted (from 2 to 5 orders of magnitude). Consequently, the lifetime of a BLE sensor operating in the connectionless mode is approximately twice as long, while the energy cost per bit of data is 2–3 orders of magnitude larger when compared to the connection-based mode. The article concludes that connectionless BLE communication is suitable for IoT applications where sensors report small amounts of data (with required throughput up to approximately 280 Bps) and have to operate on batteries for long periods of time. Unfortunately, the authors did not analyze large-scale deployments and did not conduct experiments that would validate the theoretical results.

The energy efficiency of BLE tags was thoroughly studied for various hardware architectures and application scenarios: BLE 121LR platform in the opportunistic data transmission [13], CC 2541 and nRF 51822 in the continuous monitoring application [21], nRF 51822 operating in the connectionless mode [22], and Stick’N’Find tags used as beacons in the localization system [16]. All these works report very low power consumption for the connectionless mode, that is significantly lower than the energy consumed in the connected mode. Unfortunately, none of these works have conducted experiments in large-scale deployments.

Harris et al. [20] verified, with the use of simulations (using *ns-3* simulator) and experiments, BLE efficiency in dense IoT deployments where a high collision rate and energy consumption are expected. Experimental evaluation was conducted for 10 minutes using up to 9 Nexus 5 smartphones as scanners and up to 20 Estimote tags. The focus was on active scanning, with the scanners sending scan requests, and the tags sending advertisements every 950 ms and responding with scan responses. Harris introduced two metrics: (i) X-second success, which equals 1 if in every X-second-long time window (from the initial scan request) at least one scan response is received; and (ii) total success, which is the ratio of a successful scan request–scan response interaction versus all expected interactions during the test. The total success rate achieved in [20] for a single scanner and a single advertisement device was 85% and dropped to slightly above 80% for 20 advertisers. It was shown experimentally that both the total and 5-s success rates drop significantly with the number of active scanners, while the number of advertisers was shown to be less important. Unfortunately, the authors neither verify the performance of passive scanning (when there are no scan requests and scan responses sent) nor perform experimental evaluation with a larger number of advertisers.

The article by Shan et al. [10] investigates collisions of advertisements when a large number of BLE devices is located in a narrow area. The experiments were carried out with 40 iBeacon devices using an advertisement interval above 1 s, and a single scanner built using a RaspberryPi (RPi) single board computer. The scanner operated in a passive scanning mode with a scan interval of 10.24 s. For the network of up to 10 tags, the advertisement reception rate was close to 100%, but dropped quickly for larger numbers, reaching approximately 61% for 40 tags. The scope of this work was similar to ours, but a relatively small number of tags was used in the experiments and large advertisement intervals were used. Moreover, the article also lacks more detailed information about the operation of the RPi-based scanner.

The paper by Shan and Roh [12] focuses on the discovery time of BLE tags. Their goal was to tune BLE parameters (namely, the advertisement interval) in order to shorten the detection time of BLE tags by the scanner running in a continuous scanning mode (i.e., the scan interval and the scan window parameters are set to the same value) and lower the energy consumed by the tags during discovery. The detection time investigated by Shan and Roh corresponds to the X-second success metrics. The numerical and simulation results presented in this paper show that, for an advertisement interval between 200 ms and 3 s, detection of up to 500 tags takes less than 10 s. In other words, within 10 s a single scanner receives at least one advertisement packet from each of the 500 tags—this corresponds to 10-s success metrics (for the passive scanning) which, for 500 devices, is equal to 100%. Smaller advertisement intervals (below 200 ms) and those above 3 s cause detection times to increase significantly (cf. Figure 8 in [12]). Although Shan and Roh only focused on tag discovery and analyzed BLE using just simulations, their results show that if the advertisements contain data (e.g., some measurements), then in dense environments it takes several seconds (and, consequently, transmission of several advertisements) to successfully transmit the data to the scanner. Moreover, the optimal advertisement interval should increase with the number of BLE tags, with values between 100 ms and 2 s being appropriate for networks of up to 1000 tags. Unfortunately, the article lacks the real life validation that would take into account the imperfections of real hardware and software.

The performance of data transmission in the advertisement mode depends on both the efficiency of the packet reception and the packet collisions in the communication channel. Ghamari et al. [22] examined advertisement collisions when a large number of BLE nodes simultaneously transmit data. The paper addresses energy costs incurred by the collisions and the development of the collision model, which can be used to estimate the collision ratio for a different number of advertising devices. The presented results are based on theoretical analysis and small-scale experiments with 7 tags. The results show that decreasing the advertisement intervals greatly increases the probability of packet collisions, which in turn affects the quantity of received advertisements and throughput, and also increases the energy consumed by the BLE tags. Unfortunately, the small-scale real life validation of the findings is a shortcoming of this article, since large-scale performance was only analyzed theoretically.

The previous studies on the performance of BLE are mostly based on theoretical analyses and simulations that targeted connection-based communication. Since advertisement messages are commonplace in BLE networks, it is interesting to assess to what extent they can be used for data transmission in connectionless communication. To date, only a few papers have addressed this topic and none have conducted a large-scale experimental evaluation.

## 3. Experimental Setup

The aim of our experiments was to verify if the connectionless BLE mode applies to data transmission in IoT applications. In contrast to work previously published by other authors, we chose to run a real life experiment with a large number of devices in order to provide an insight into how such networks operate in practice. The experiments allowed the analysis of BLE operation using real, imperfect transceivers (which are often used in IoT systems) operating in the presence of interference from other BLE devices and other 2.4 GHz networks.

In the experiments, we imitated one of the most typical IoT applications: distributed monitoring. In this application, the tags are deployed in a small or medium size area, and possibly move around at low speeds. A real-life example of such an application is the well-being monitoring of animals on a farm. In such an application, tags are worn by the animals and hubs are deployed in the sheds. For dairy cows, for example, roughly 100 cows live in one shed with an area of approximately 1000–1500 $m^{2}$. For smaller animals, for example sheep or goats, the number of animals (tags) in the shed can significantly grow. During the operation, the tags perform measurements, data processing, and aggregation, and send the resulting information to the infrastructure devices (hubs) deployed in the area. The hubs aggregate the received information and forward it to the central server for further processing and storage. Communication from the tags to the hubs uses BLE advertisements. Other technologies (e.g., WiFi, Ethernet, GSM) are used to link the hubs with the server.

**Figure 2:**
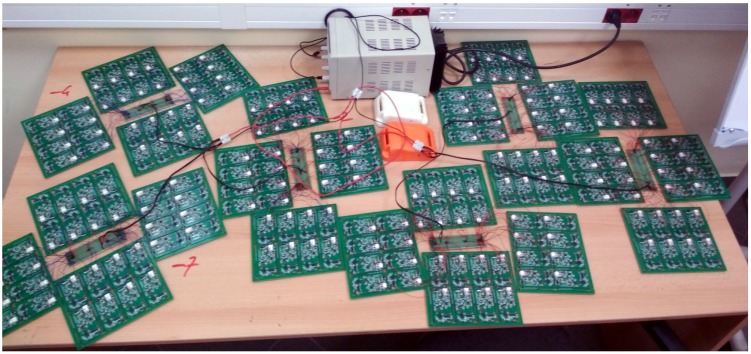
Photograph of the BLE tags deployed in the laboratory.

The abovementioned scenario was reproduced in an experimental setup at the university (Figure 2). The number of tags was 210, we used one hub, and deployed all the devices in a laboratory (roughly 21 $m^{2}$).

### 3.1. Hub

**Figure 3:**
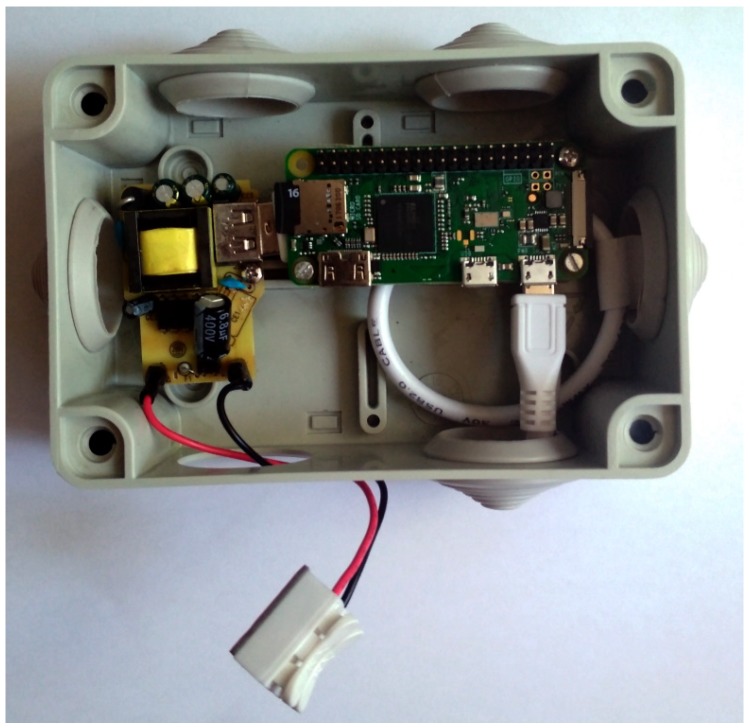
Photograph of the hub used as the scanner in the experiments.

The hub is based on the RPi Zero W single board computer (Figure 3). This version of RPi is compact and includes a WiFi- and BLE 4.1-compatible radio, which makes it a perfect candidate for our application.

The RPi runs a Linux operating system with BlueZ 5.50, an official Linux Bluetooth protocol stack. The hub runs a Python script that starts a BLE sniffer and uses the MariaDB database management system for storing configuration parameters and results. Upon startup, the sniffer loads a list of the tag’s MAC addresses and starts a passive scanning, i.e., no scan request–scan response messages are exchanged. The list is used for filtering the incoming BLE packets—only packets originating from the tags on the list are analyzed and stored in the database (we did not use the whitelisting feature from the BLE specification due to the limited size of the whitelist). During the operation, the sniffer continuously scans for advertisements, processes them in Python script, and then stores information in the database. The stored information includes the message’s payload, the tag’s MAC address, the received signal strength indicator (RSSI), and timestamp. To speed up the operation, the database contains a single table without indexes and constraints.

We decided to build the hub using a general purpose single board computer. The goal was to verify if an off-the-shelf, low-cost, and easy to use device can be used as a hub in real life for a BLE-based IoT application. As the hub is not a dedicated device, its underperformance in receiving and processing the advertisements (resulting from receiver saturation, communication and processing delay, channel switching delay, etc.) may adversely affect the results of the evaluation. This does not however impair verification if the BLE connectionless mode can be used in large-scale IoT applications. Nevertheless, the performance of the hub, when advertisements from a few tags are received, should still be estimated. This was the goal of the first evaluation scenario (Section 3.3) and allowed us to assess the relative performance drop in subsequent scenarios.

### 3.2. Tags

The tags are dedicated BLE devices designed and developed for the purpose of a distributed monitoring application. The device is based on the nRF 52832 system-on-chip from Nordic Semiconductor, which incorporates a BLE-5.0-compatible radio and ARM Cortex-M4F microcontroller. Additional components on the device include sensors and power circuits.

In the target application, the tags continuously take the sensors’ readings, perform data analysis and aggregation, and periodically broadcast scannable undirected advertisement messages. The advertisement interval equals 250 ms, which is a trade-off between packet collision probability and the effective network throughput [22]. Message payload changes every *data interval* ($T_{\mathrm{DI}}$) and contains the successive portion of the collected measurements. During the lab experiment, the advertisement payload did not contain actual data, but a sequence number and random data up to the total length of 30 bytes. The goal of the target application is to ensure that all the measurements are successfully transmitted from the tags to the hub. In the experiments, the sequence numbers were used to simulate distinct measurements and validate if every measurement successfully reaches the hub. For successful transmission, it is enough that at least one advertisement for each sequence number is correctly received. As the sequence numbers change every 10 s (i.e., $T_{\mathrm{DI}}$ = 10 s) and the advertisement interval equals 250 ms, upon successful transmissions the hub should receive 40 advertisements with the same sequence number—in fact, the expected number of advertisements per sequence number is lower, as the advertisement interval is biased with a random delay of between 0 and 10 ms. The shift is evenly distributed, so on average it is equal to 5 ms. The expected advertisement interval equals 255 ms and the expected number of advertisements per sequence number is 39.

### 3.3. Scenarios

The laboratory experiments used one hub to monitor two tags (referred to as A and B) randomly selected from all the available tags. Other tags (up to 210 tags) were used to generate background interference and collisions in the communication channel. These devices were under full control regarding their mode of operation and transmission parameters (e.g., frequency, power, payload length). As the experiments were carried out in the university laboratory, external BLE devices (e.g., owned by students) might have appeared randomly in the vicinity and could have introduced additional variance in the measurements. These devices were out of our control and may have affected the results of the experiments. It is worth mentioning that such a situation is beneficial as it is common to real life applications, when one cannot have full control over the environment and external devices operating in the vicinity. To assess and minimize the influence of such devices, the tests were conducted over a long period of time, both during the day and night. As presented in Section 4.2, the external devices have a small influence on the experiments.

During the experiment, all the tags (including tags A, B, and all interference tags) were transmitting advertisements with a transmission power of 0 dBm and a payload length of 30 bytes. Tag A and the interfering tags used an advertisement interval equal to 250 ms, and tag B had the advertisement interval set to 750 ms. A different interval was selected for tag B in order to investigate which interval better suites the target application in the presence of heavy interference. The transmitted payloads contained random data and sequence numbers that changed every 10 s ($T_{\mathrm{DI}}$ = 10 s). The maximal number of advertisements received for each sequence number from tag A is ${\mathrm{ADV}}_{A}$ = 39 and for tag B, ${\mathrm{ADV}}_{B}$ = 14.

Scenario 1 was run to evaluate the performance of the RPi as a scanning device when only tags A and B were transmitting. This scenario was run for 68,996 s, which is approximately 19 h and 10 min (Table 1). The purpose was to investigate how many advertisement messages are correctly received, processed, and stored by the hub, and what is the expected ratio of missing messages due to underperformance of the RPi.

The goal of Scenario 2 was to evaluate how reception of the advertisements drops as a function of the number of tags simultaneously transmitting when only tags A and B were recorded by the hub. The greater number of transmitting tags corresponds with a higher likelihood of packet loss due to collisions in the communication channel, as well as the higher load of the hub that is needed to filter out advertisements (except those coming from tag A and B). Scenario 2 was run for 76,525 s, which is approximately 21 h and 15 min.

In Scenario 3, we tested decreases in performance when a large number of tags sent their advertisements simultaneously, with a big number of them being recorded by the hub. This scenario shows how the performance of the hub drops with an increasing workload (i.e., the number of tags for which advertisements are processed and stored in the database). The experiment in this scenario was run for 11,749 s, i.e., slightly over 3 h and 10 min.

In all the scenarios, we collected raw advertisements and calculated several metrics to analyze performance:

- Min, max, and average number of advertisements received per each sequence number;
- Minimal number of advertisements ($A_{99\%}$) so that 99% of all sequence numbers were received with $A_{99\%}$ or more advertisements (in other words, 1% of sequence numbers were missed or received with a smaller number of advertisements than $A_{99\%}$);
- Number of missing sequence numbers ($S_{missing}$);
- $T_{\mathrm{DI}}$ second data reception rate (${\mathrm{DRR}}_{T_{\mathrm{DI}}}$), calculated as the ratio between the number of sequence numbers received by the hub and the number of sequence numbers (iterated every $T_{\mathrm{DI}}$ data interval) transmitted by the tag;
- Packet delivery rate (PDR)—the ratio between the number of advertisements received by the hub (and stored in its database) and transmitted by the tag. This ratio takes into account the losses of the radio packets due to collisions in the communication channel as well as the underperformance of the hub (e.g., losses due to receiver saturation, buffer overflows, etc.).

The above definition of ${\mathrm{DRR}}_{T_{\mathrm{DI}}}$ is similar to the X-second success defined in [20], but we calculated it for the sequence numbers transmitted in the advertisement messages rather than the scan response messages. ${\mathrm{DRR}}_{T_{\mathrm{DI}}}$ equals 100% if at least one advertisement for each sequence number is correctly received for sequence numbers that change every $T_{\mathrm{DI}}$ seconds. As a result, DRR can be high (close to 100%) even if PDR is low for an appropriately chosen $T_{\mathrm{DI}}$ interval.

For an application that uses connectionless BLE communication, it is important to maintain a high DRR. In dense environments, PDR would inevitably be small, but DRR can be kept high by properly setting the advertisement interval and data interval. These parameters determine the number of advertisements containing the same payload (data), and thus the effective DRR for the given PDR. Therefore, the anticipated value of DRR can be close to 100%.

## 4. Results and Discussion

### 4.1. Results

In Scenario 1, only tags A and B were transmitting. The number of received advertisement messages per single sequence number, from tag A, varied between 10 and 36 with 25 advertisements being the average.

**Figure 4:**
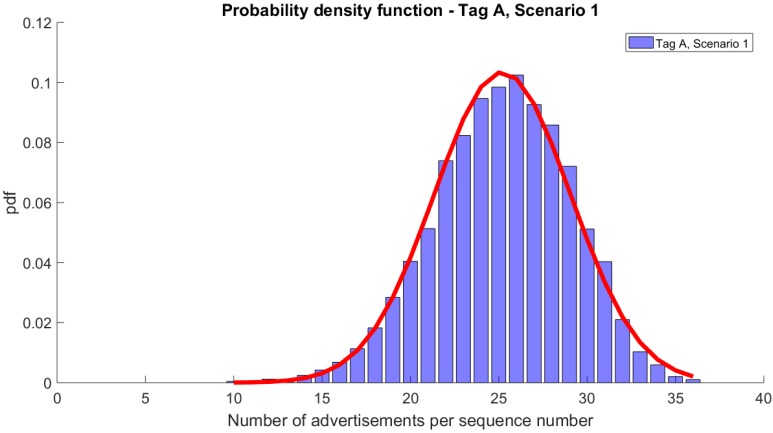
Probability density function of the number of advertisements per sequence number for tag A and its approximation with Normal distribution.

Figure 4 shows the probability density function (pdf) of the number of advertisement messages per sequence number. This pdf follows Normal distribution with μ = 25.2 and σ = 3.9.

**Figure 5:**
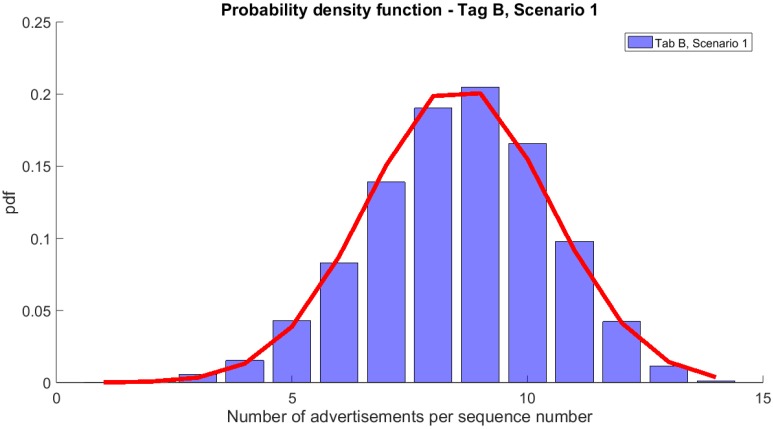
Probability density function of the number of advertisements per sequence number for tag B and its approximation with Normal distribution.

As presented in Figure 4 and Table 2, from the expected 39 advertisements, at most 36 were correctly received. In the worst case, the single sequence number was received in 10 advertisement messages. Over 99% of sequence numbers ware correctly received in 17 or more messages, which is approximately half of the expected 39 (only 1% of unique sequence numbers were received in less than 17 advertisements). In the same scenario, tag B (advertising every 750 ms) successfully transmitted up to 14 advertisements per sequence number (this is equal to the expected number), however for some of the sequence numbers the number of advertisements was as low as one (Figure 5). Still, all sequence numbers in the experiment were successfully received by the hub and 99% of the numbers were received at least 4 times.

It is worth mentioning that although ${\mathrm{DRR}}_{10s}$ (i.e., the data reception rate when $T_{\mathrm{DI}}$ = 10 s) equals 100% for both tags in this scenario, PDR equals 64% and 60% for tag A and B, respectively. As only two tags were transmitting, we can assume that the collisions are negligible and that the 40% loss in PDR is due to the underperformance of the RPi that was used as the hub.

In Scenario 2, the packet reception ratio dropped, but it still ensured a very high ${\mathrm{DRR}}_{10s}$ and met the requirements of the application. For tag A, all sequence numbers were correctly received (${\mathrm{DRR}}_{10s}$ = 100%), however some in only 3 advertisement messages (out of 39 transmitted). For tag B, some sequence numbers were missing (${\mathrm{DRR}}_{10s}$ = 99.9%) and the average number of advertisement packets per sequence number dropped by 25%. For both tag A and B, the packet reception rate dropped by approx 22% when compared to Scenario 1.

The number of advertisements received per sequence number dropped even more in Scenario 3. Although all sequence numbers were received from tag A, some of them were received in only 2 messages (compared to 10 in Scenario 1) and the average number of advertisements per sequence number dropped from 25 to 10. For tag B, 10 (out of a total of 1175) sequence numbers were not received. For those that were received, only a few were received 9 or 10 times. In fact, most of the sequence numbers were successfully transmitted in 3 or 4 advertisements, which is a significant drop when compared to the initial 8 and 9 times in Scenario 1. Although obtaining the worst PDR (below 30%), both tags A and B were able to meet the requirements of the foreseen monitoring application with ${\mathrm{DRR}}_{10s}$ being above 99%.

### 4.2. Discussion

**Figure 6:**
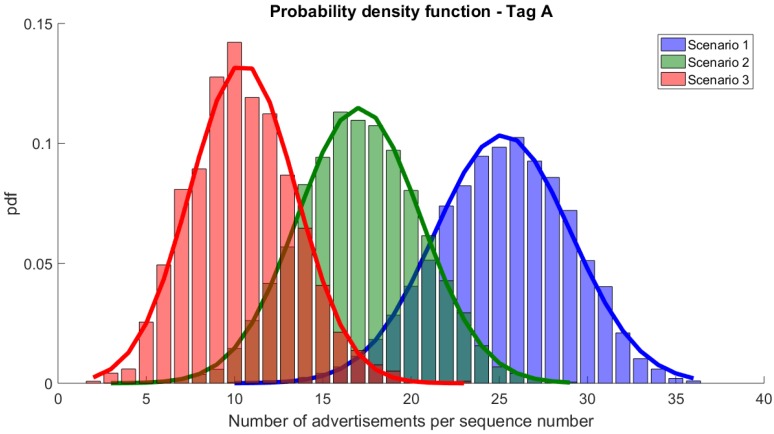
Probability density function of the number of advertisements per sequence number for tag A and its approximations with Normal distributions.

**Figure 7:**
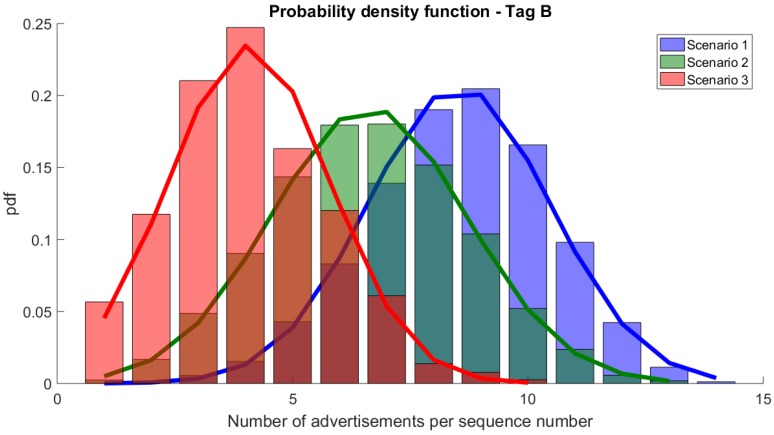
Probability density function of the number of advertisements per sequence number for tag B and its approximations with Normal distribution.

Figure 6 compares the pdfs for tag A and Scenarios 1, 2, and 3. As presented, the numbers of advertisements per sequence number drop with the number of transmitting devices and the workload of the hub. Approximation with Normal distribution is accurate. A similar drop can be seen for tag B (Figure 7).

Table 2 presents the statistics of advertisements per sequence number (min, average, max, 99%, missing sequence numbers) as well as the PDR and DRR for all the scenarios and for both tags A and B. The efficiency of advertisement transmission (PDR) is moderate (slightly above 60%), even for the first scenario where there was no interference from other BLE tags. As interference increased (Scenario 2), PDR drops to below 50%. The further drop (Scenario 3) is probably due to the underperformance of the hub that was processing advertisements from 52 distinct tags. The low PDR in Scenario 1, and the significant drop in Scenario 3 compared to Scenario 2, shows that the efficiency of the hub is important for overall performance. The performance drop between Scenario 1 and Scenario 2 is mostly due to interference and collisions caused by all the BLE tags transmitting simultaneously with tag A and B. From the drop of PDR values, we can infer the probability of the collision in the communication channel when 210 tags are advertising. It turns out that collision probability is equal to approximately 0.33 for tag A and 0.22 for tag B. These values are quite similar to the theoretical and simulation results presented in [22], where the probability of collision for 200 tags and advertisement intervals of 200, 300, 700, and 1000 ms was estimated at approximately 0.51, 0.4, 0.22, and 0.15, respectively.

**Figure 8:**
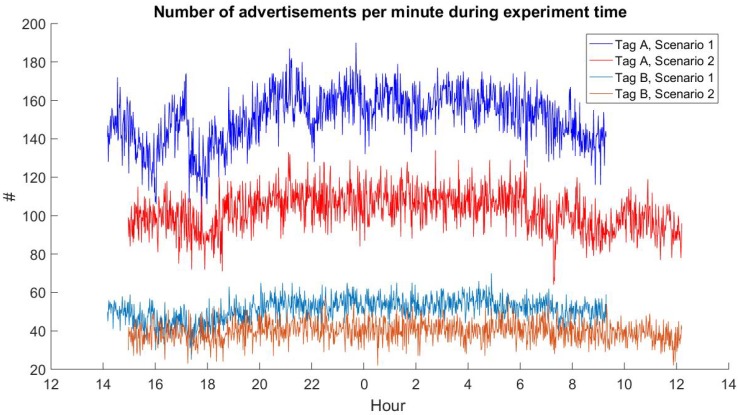
The number of advertisements received per minute during the experiment in Scenarios 1 and 2.

Figure 8 presents the number of advertisements received per minute (over the time of the day) from tag A and tag B in Scenario 1 and Scenario 2 (Scenario 3 lasted less than 4 h and was thus omitted). It can be noted that the number of received advertisements increased during the night, and that the variance of this number is also smaller at night—from approximately 8 pm to 6 am the next day. This trend is quite clearly visible for tag A and can also be noticed for tag B in Scenario 1. For tag B, in Scenario 2, the day/night change in the number of advertisements per minute is not visible, which is probably due to the low number of advertisements received in this scenario from tag B. Although this would require additional investigations, we think that the improved performance at night time might be the result of lower collisions from external BLE devices and WiFi networks operating in the building and sharing the same radio spectrum. The change in the performance is small, thus confirming our assumption that external devices will not significantly affect the results of our experiments.

**Figure 9:**
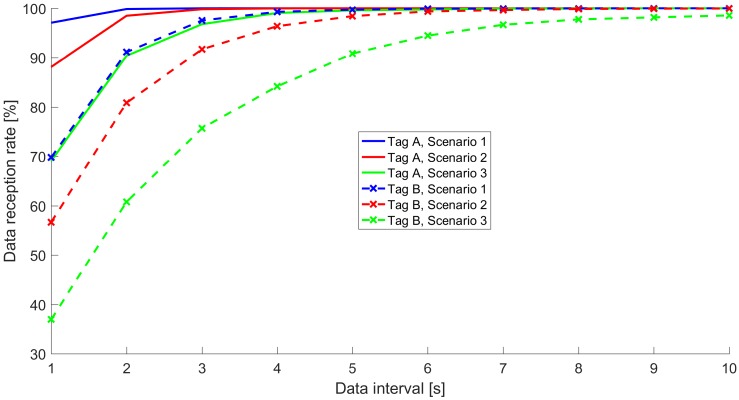
Data reception rate for different data intervals.

Figure 9 presents the data reception rate parameter for the cases in which advertisement payload changes at a different data interval than that used in the experiments (namely, $T_{\mathrm{DI}}=1,2,\ldots ,9$ s). The reported values were calculated based on timestamps recorded by the hub with each advertisement received during the experiment. The data reception rate inevitably decreases as the data interval shortens, but for tag A, it was above 99% for $T_{\mathrm{DI}}\geq 4$ s in all the Scenarios. For tag B, the data reception rate goes below 99% for data intervals smaller than 8 s and 10 s in Scenarios 2 and 3, respectively, but for the data interval greater or equal to 5 s it is always above 90%. This shows that conectionless BLE communication can ensure high data reception rates, even for data that changes quite frequently. As a result, BLE can be used in time-constrained IoT applications where LPWAN technologies can not be used due to duty-cycling and message frequency restrictions.

**Figure 10:**
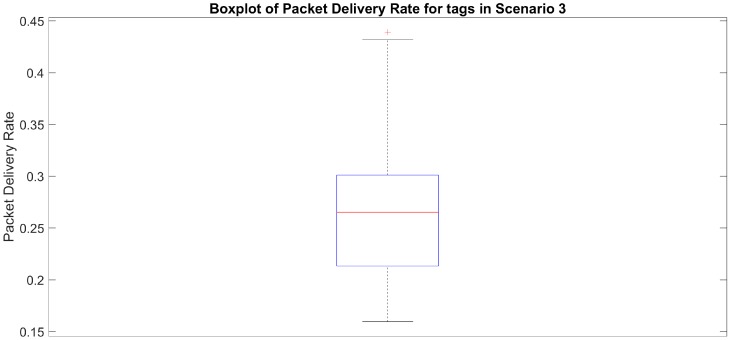
Boxplot of the packet delivery rate (PDR) for 52 tags recorded in Scenario 3.

Figure 10 presents PDR for all the tags that were recorded by the hub in Scenario 3 (i.e., 52 tags each sending advertisements every 250 ms). As can be seen, the reception rates vary significantly, for some of the tags reaching almost 44%, while dropping below 20% for others. Most of the tags recorded had PDR in the range between 21% and 30% (lower and upper quantile), with the median equal to 26%. This suggests that some of the tags were performing worse when compared to tag A. However, it can also be the case that the variations in PDR could have been a consequence of the deployment of nodes during the test—the distance from the tags to the hub was not equal, and their respective spatial placement and relative orientation was also different, which might have had an effect on radio propagation and reception.

**Figure 11:**
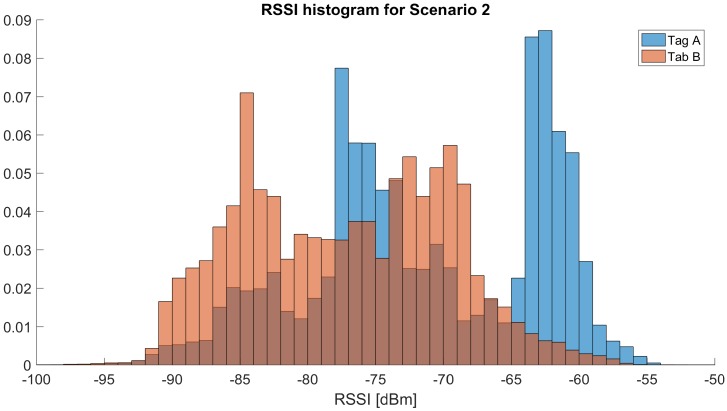
Histogram of the received signal strength indicator (RSSI) for tags A and B in Scenario 2.

The RSSI values measured by the hub for all the tags and scenarios have great variability, with results ranging from −55 to −96 dBm. The experiments show, for all the monitored tags and scenarios, that the RSSI histograms are multimodal with two or three distinct peaks (Figure 11). Our hypothesis is that this results from the three different radio channels used for sending the advertisements. However, because this characteristic of RSSI does not affect our analysis and result, further investigation is out of scope of this article.

Moreover, there is a safe margin between the lowest received RSSI and the sensitivity level of the hub (equal to −97 dBm). This suggests that advertisement losses (low PDR values) are due to collisions in the communication channel and underperformance of the hub, rather than attenuation of the radio signal. It also shows that tags can safely lower the transmission power by several dB in order to preserve energy without degenerating PDR.

Even though power consumption and energy efficiency were beyond the scope of our experiments, we still performed a basic evaluation and analyses. For the advertisement interval of 250 ms, the tags consumed 227 μA on average at a supply voltage of 3.6 V. For the advertisement payload containing 30 bytes of data, which changed every $T_{\mathrm{DI}}$ = 10 s, this yields an energy cost of 8.172 mJ or 32.9 μJ/bit. These values are comparable to the measurements reported in other articles (e.g., [13,16,22]). A further reduction in the energy consumption can be achieved by adjusting the tag’s operation, e.g., lowering the transmission power, decreasing the frequency of the advertisements [13,22], or switching the tag to sleep mode after a predefined number of advertisements is sent.

## 5. Conclusions

This paper presented experimental evaluation of BLE advertisement communication when a large number of advertisers are deployed in a small area. Such scenarios are representative to a wide range of IoT applications. The experiment confirms a previous analytical and simulation evaluation that over 200 BLE advertisers can be used to successfully transmit data simultaneously (DRR > 99%), despite collisions. For larger networks, the probability of packet collision would increase [22], therefore, in order to sustain a high data reception rate, the tag and the hub parameters (advertisement interval, scan interval, scan window) need to be adjusted and the data interval $T_{\mathrm{DI}}$ needs to be increased. These changes will however lower the effective data throughput.

As BLE has no duty-cycling limits, and that in advertisement mode data is transmitted periodically, one can achieve a high DRR with the appropriate selection of the advertisement interval and data interval. As presented, for an advertisement interval of 250 ms and a data interval greater then 4 s, DRR is greater than 99%. When advertisements contain 30 bytes of information, the effective transmission data rate is approximately 8 Bps. Although small, it allows 28 kB of data to be transmitted per hour, which is enough for various IoT applications. Simply consider LoRa, one of the most promising examples of unlicensed sub-GHz technology. With a spreading factor of 12,250 kHz bandwidth, and the same payload size, transmission takes approximately 700 ms. For 1% duty-cycling (the widely used setting in sub-GHz networks), this translates to 51 transmissions or 1.5 kB of data per hour. Even for lower spreading factors of 7 and 9, the amount of data transmitted per hour may increase to approximately 16 and 5 kB respectively, but at the same time the effective communication range drops significantly.

Based on the experimental results, we argue that the BLE connectionless mode is well suited for IoT applications, especially for those that operate in small or medium areas, indoors, or require higher throughput when compared to sub-GHz radio technologies. A lack of duty-cycling restrictions, low energy consumption, a long expected lifetime, and good coexistence when deployed in mass scale make BLE technology an attractive alternative to other radio technologies. Additional features of BLE technology, such as advertisement extensions, optional scan request/response communication, the possibility to communicate in connected mode, and the ability to estimate the angle-of-arrival, further extend the range of possible BLE applications to IoT systems.

The conducted experiments show that advertisement-based BLE communication scales well and can be used in real-life IoT applications. Future investigations may include the assessment of energy consumed by the tag (as a function of its parameters and the number of simultaneously communicating tags), and a more detailed evaluation of the impact of external advertisers on the performance of the system.

## Figures and Tables

**Table 1:** Evaluation scenarios and their parameters.

Scenario	# Tags Transmitting	# Tags Recorded	Duration	# Sequence Numbers Sent
1	2	2	19 h 10 min	6900
2	210	2	21 h 15 min	7652
3	210	52	3 h 10 min	1175

**Table 2:** Statistics for tag A and B for the different scenarios.

Metrics	Tag A Scenario			Tag B Scenario		
	1	2	3	1	2	3
$A_{\min}$	10	3	2	1	0	0
$A_{\mathrm{avg}}$	25	17	10	8	6	4
$A_{\max}$	36	29	23	14	13	10
$A_{99\%}$	17	9	4	4	2	1
$S_{missing}$	0	0	0	0	5	10
μ	25.2	17.1	10.3	8.5	6.6	4
σ	3.9	3.5	3	1.9	2.1	1.7
${\mathrm{DRR}}_{10s}$	100%	100%	100%	100%	99.9%	99.1%
PDR	64%	43%	26%	60%	47%	29%
